# Non-Destructive and Rapid Variety Discrimination and Visualization of Single Grape Seed Using Near-Infrared Hyperspectral Imaging Technique and Multivariate Analysis

**DOI:** 10.3390/molecules23061352

**Published:** 2018-06-04

**Authors:** Yiying Zhao, Chu Zhang, Susu Zhu, Pan Gao, Lei Feng, Yong He

**Affiliations:** 1 College of Biosystems Engineering and Food Science, Zhejiang University, Hangzhou 310058, China; zhaoyy@zju.edu.cn (Y.Z.); chuzh@zju.edu.cn (C.Z.); sszhu@zju.edu.cn (S.Z.); lfeng@zju.edu.cn (L.F.).; 2 Key Laboratory of Spectroscopy Sensing, Ministry of Agriculture, Zhejiang University, Hangzhou 310058, China; 3 College of Information Science and Technology, Shihezi University, Shihezi 832000, China; gp_inf@shzu.edu.cn; 4 State Key Laboratory of Modern Optical Instrumentation, College of Optical Science and Engineering, Zhejiang University, Hangzhou 310058, China

**Keywords:** hyperspectral imaging technique, single grape seed, principal component analysis, support vector machine, discrimination and visualization

## Abstract

Hyperspectral images in the spectral range of 874–1734 nm were collected for 14,015, 14,300 and 15,042 grape seeds of three varieties, respectively. Pixel-wise spectra were preprocessed by wavelet transform, and then, spectra of each single grape seed were extracted. Principal component analysis (PCA) was conducted on the hyperspectral images. Scores for images of the first six principal components (PCs) were used to qualitatively recognize the patterns among different varieties. Loadings of the first six PCs were used to identify the effective wavelengths (EWs). Support vector machine (SVM) was used to build the discriminant model using the spectra based on the EWs. The results indicated that the variety of each single grape seed was accurately identified with a calibration accuracy of 94.3% and a prediction accuracy of 88.7%. An external validation image of each variety was used to evaluate the proposed model and to form the classification maps where each single grape seed was explicitly identified as belonging to a distinct variety. The overall results indicated that a hyperspectral imaging (HSI) technique combined with multivariate analysis could be used as an effective tool for non-destructive and rapid variety discrimination and visualization of grape seeds. The proposed method showed great potential for developing a multi-spectral imaging system for practical application in the future.

## 1. Introduction

Grape (*Vitis vinifera* L.) is widely cultivated throughout the world. The planting area exceeds 7.5 million hectares with a global production of 75.1 million tonnes [1]. An enormous amount of grape pomace, which mainly consists of grape seeds and skins [2], is generated from the winemaking industry and juice production worldwide. Grape pomace has gained attention for its high content of bioactive phenolic compounds, and recently, it has been increasingly used as a large natural supplement of antioxidants in food and drug, health product and cosmetic industries [3]. Grape seeds are particularly rich in phenolic compounds. Though grape seeds account for only 0–6% of berry weight, they contain about 20–55% of the total phenolic compounds of the grape berry [4,5]. The grape nutritional qualities are affected by various environmental conditions, including climatic and geographical factors, viticultural and post-harvesting practices, as well as the degree of maturation, and variety is the determinant factor resulting in variation [6,7,8]. Some studies have investigated the variation of chemical composition and antioxidant properties in grape seeds among different varieties [8,9]. Grape seeds of a particular variety with a high content of phenolic compounds are beneficial for producing antioxidant products. Full utilization of such a variety of grape seeds contributes to lower production cost of antioxidant products and less environmental pressure. Therefore, searching for appropriate methods for variety discrimination of grape seeds is of great importance.

Visual inspection is the simplest way of seed characterization. It can be easily performed without the aid of any instruments. However, this method is time-consuming, laborious, subjective and requires professional knowledge. Recently, some other approaches such as morphological analysis [10] and molecular markers [11] have also been developed. These methods are destructive and time-consuming, which hinders their on-line and large-scale application in practice. Thus, there is a growing demand for developing non-destructive and rapid methods. Hyperspectral imaging (HSI), as a promising technology for non-destructive and rapid measurement, has gained much attention in different fields. HSI integrates machine vision and spectroscopy technology in one system and can provide spatial and spectral information simultaneously [12]. Unlike near-infrared (NIR) spectroscopy, HSI collects the detailed pixel-wise spectra of the samples and records the image information of the original location at the same time. It has the potential of presenting the spatial distribution of the physicochemical properties within each sample, as well as among different samples [13]. The characteristics of batch detection extend its application in the modern seed industry. Recently, HSI has been used in quality assessment of agricultural seeds, such as for variety and geographical origin identification [14,15], viability and vigor prediction [16], bacterial and fungal infection [17,18], etc. As for variety discrimination, the number of seeds should be sufficient to contain a broad variation, which will consequently result in high computational cost. For real-world application of an HSI technique, the robustness and reliability of modelling are crucial. Therefore, multivariate analysis methods, including preprocessing, variable extraction and modelling methods, are applied in the data processing of hyperspectral images. With the aid of multivariate analysis methods, the number of samples can be significantly extended for analysis.

The main objective of this study was to explore the feasibility of using an HSI technique in the NIR spectral region of 874–1734 nm for variety discrimination and visualization of grape seeds. The specific objectives were to: (i) form the scores of the images with principal component analysis (PCA) to recognize the patterns among the three varieties, (ii) form the PCA loading plots to identify the effective wavelengths (EWs), (iii) build the discriminant model by support vector machine (SVM) and (iiii) assess the developed methods by predicting and visualizing the external validation image of each variety.

## 2. Results

### 2.1. Overview of Spectral Profiles

**Figure 1:**
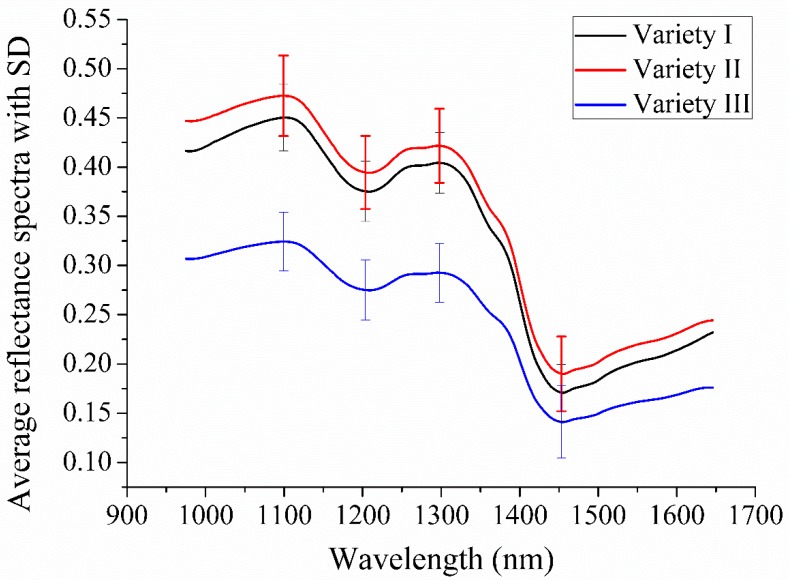
The average spectra of grape seeds of three varieties in the range of 975–1646 nm. SD: standard deviation.

Figure 1 shows the average spectra of grape seeds of three varieties with the spectral standard deviation (SD). The trends of the average spectra of three varieties were similar. The major spectral bands for all three varieties were identified around 1100, 1200, 1300 and 1450 nm. However, due to the different compositions and physicochemical characteristics of these three varieties, differences related to spectral features existed in the reflectance values. The spectral peaks and valleys occurring in the NIR spectral region are primarily related to overtones and combinations of fundamental vibrations of C–H, N–H and O–H groups [19]. The spectral bands between 1100 and 1390 nm are associated with the second overtone of C–H stretching [20]. The spectral valley around 1450 nm may be attributed to the first overtone of O–H stretching [21]. Especially, overlaps in SD at the four spectral bands of Varieties I and II could be observed, owing to the similar chemical composition of these two varieties. In addition, reflectance values between Varieties I and II in the spectral curves within the range of 1300–1400 nm were close, which mainly corresponded to the combination bands of C–H vibrations [12].

### 2.2. Principal Component Analysis

**Figure 2:**
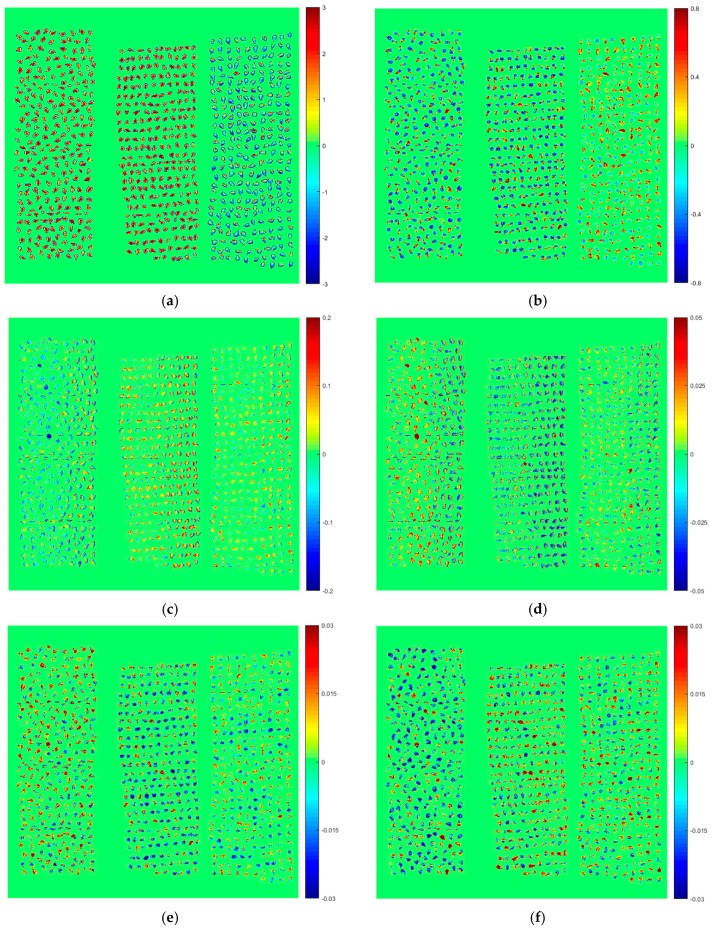
Scores images of the first six principal components (PCs) for: (**a**) PC1; (**b**) PC2; (**c**) PC3; (**d**) PC4; (**e**) PC5; and (**f**) PC6.

Principal component analysis (PCA) was firstly conducted on the hyperspectral images to explore the differences among the three varieties. For each variety, PCA was performed on a randomly chosen hyperspectral image. The first six principal components (PCs) explained 95.70%, 4.04%, 0.18%, 0.04%, 0.02% and 0.01% of the total variance, respectively. That is to say, the total variance explained by the first six PCs was 99.99%. These six PCs showed the most significant variation among samples, and the scores of sample pixels were obtained for each PC. By multiplying the scores of the first six PCs and the corresponding binary value of each pixel in the mask (zero as the background pixels and one as the sample pixels), score images were formed and visualized by using a color bar (Figure 2a–f). It was comparatively intuitive to make a distinction between samples of Varieties I and II and samples of Variety III from Figure 2a,b,d,e. Grape seeds of Varieties I and II were clustered with high positive scores of PC1, while grape seeds of Variety III were clustered with negative PC1 scores. In addition, for PC2, PC4 and PC5 score images, absolute values of scores of most sample pixels of Varieties I and II were higher than those of Variety III, which made the color deeper. Furthermore, for PC3 and PC6, samples of Variety I and samples of Variety II and III could be roughly distinguished. However, for qualitative identification of each single grape seed, discriminant models were needed.

### 2.3. Effective Wavelength Selection

**Figure 3:**
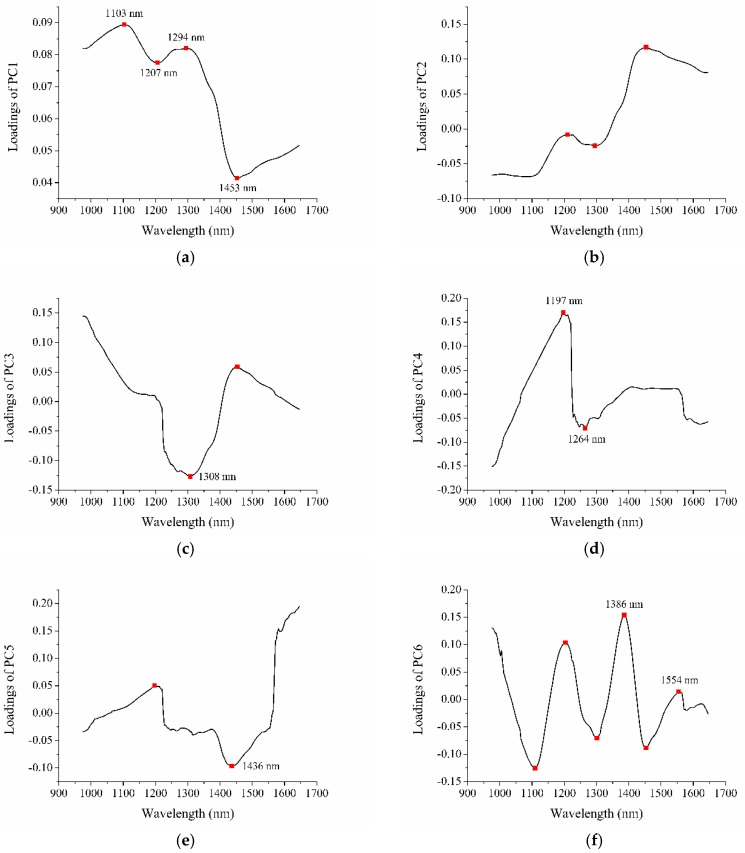
Loadings of the first six PCs for: (**a**) PC1; (**b**) PC2; (**c**) PC3; (**d**) PC4; (**e**) PC5; and (**f**) PC6.

The final sample spectra were high dimensional data. The contiguous wavelengths were highly correlated and might contain redundant information. Thus, it was vital to apply appropriate methods to extract the most informative variables/wavelengths. The variance of the first six PCs added up to 99.99%, which accounted for most of the variance in the spectral data. Thus, loadings of the first six PCs were used for variable extraction. Figure 3a–f shows the loadings of the first six PCs, respectively. It is worth noting that the trend of the PC1 curve was similar to the average spectra of grape seeds, while the curve trends from PC2–PC6 were not. Wavelengths located at the peaks and valleys of loadings curves were selected as the effective wavelengths (EWs). Some wavelengths chosen from different PCs were contiguous or very close and might have contained redundant information. Only one wavelength in the close region was chosen. In total, 10 EWs (1103, 1197, 1207, 1264, 1294, 1308, 1386, 1436, 1453 and 1554 nm) were selected. The selected EWs between 1100 nm and 1390 nm correspond to the second overtone of C–H stretching [20]. Besides, the spectral bands at 1436 nm and 1453 nm are associated with the first overtone of O–H stretching [12,21]. The spectral band at 1554 nm is the first overtone region [22]. The effectiveness of wavelength selection was then evaluated by building SVM models using these EWs.

### 2.4. Discriminant Models Based on the Effective Wavelengths

After selecting the EWs, the dimension of the calibration spectral data was reduced from 28,290 samples × 200 wavelengths to 28,290 samples × 10 wavelengths, which accelerated the modelling process. The category values of Variety I, Variety II and Variety III were assigned as 1, 2 and 3, respectively. The spectra of each single grape seed were calculated by averaging the pixel-wise spectra within the grape seed to represent the sample. The spectra based on the EWs were then extracted. The category vectors were used as the dependent variables (*Y*), and the spectra based on the EWs were used as the independent variables (matrix *X*) to develop the SVM model. The performance of the model was evaluated by the calibration and prediction accuracy. The results of the discriminant model are shown in Table 1. For the SVM model in this study, parameters, such as the penalty coefficient (c) and kernel width (g), were optimized by a grid-search procedure, with the range of *c* and *g* from 2^−8^–2^8^. The best (c, g) of the SVM model was (256, 147.0334). The SVM model obtained good results, with a calibration accuracy of 94.3% and a prediction accuracy of 88.7%. Especially, the discriminant performance of samples of Variety III was quite satisfactory, with the calibration and prediction accuracy exceeding 99%. For samples of Varieties I and II, misclassification phenomenon could be observed, owing to the similar chemical composition of these two varieties.

### 2.5. Varieties’ Classification and Visualization of External Validation Images

**Figure 4:**
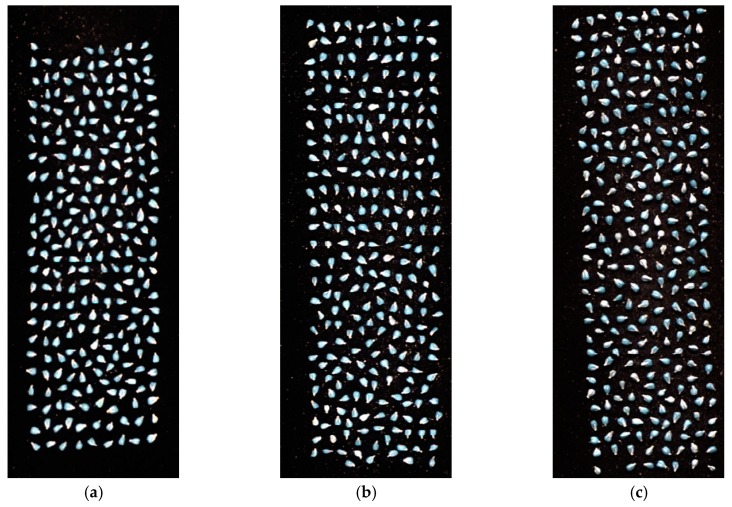

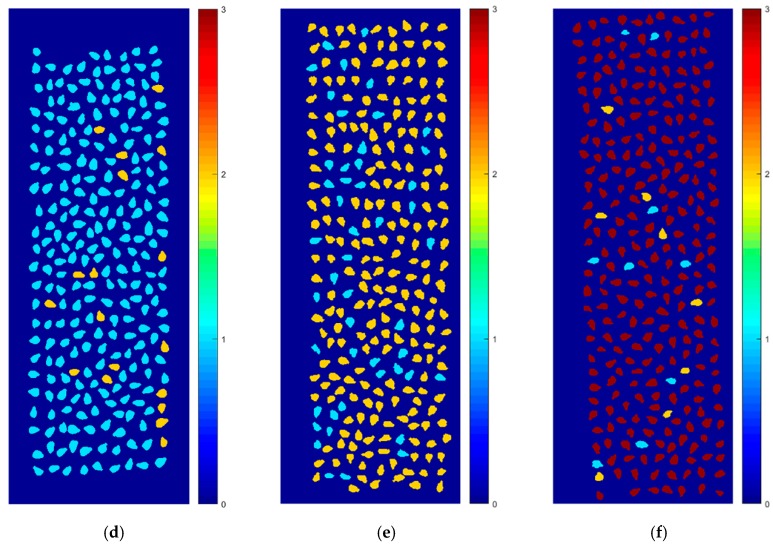
The original grayscale images of (**a**) Variety I; (**b**) Variety II; (**c**) Variety III and the corresponding classification maps of (**d**) Variety I; (**e**) Variety II; (**f**) Variety III.

External validation was applied to assess the effectiveness of the SVM model. For each variety, samples in one image that did not belong to the prediction set were used for external validation and prediction visualization. With the characteristics of acquiring spatial and spectral information together, the established SVM model could be applied to hyperspectral images to form the classification maps to visualize the spatial distribution of seed variety. The original grayscale images are shown in Figure 4a–c, and the classification maps are displayed in Figure 4d–f, respectively. The prediction accuracy of the three images was 92.9% (237/255), 82.0% (219/267) and 93.9% (261/278), respectively. The visualization results suggested that the HSI technique provided a much simpler and intuitive way to detect the grape seed variety rapidly and non-destructively than conventional methods with less consumption of time and manpower.

## 3. Discussion

A hyperspectral imaging system in the NIR region (874–1734 nm) was used to discriminate grape seeds of different varieties. The difficulty of using HSI for real-world and large-scale application lies in sample acquisition with a wide range of variation. In a previous study, characterization of grape seeds according to varieties and stage of maturation by HSI was reported [23]. A total of 56 samples (100 seeds per sample) including two red grape varieties (Tempranillo and Syrah) and one white variety (Zalema) sourced from two kinds of vineyard soil were analyzed in [23]. Average spectra of each sample were calculated, resulting in 56 spectra. In our study, hyperspectral images of over 40,000 grape seeds were collected to cover more variation. Sample spectra were extracted from each single seed, and this came with enlargement of spectral data and computational cost. Selection of the EWs significantly reduced the analysis procedure. The discriminant model based on the EWs obtained satisfactory results, which indicated the potential of dealing with massive samples and could be used for large-scale detection. Besides, acquiring images at the selected EWs would save much time for data processing and could help to establish a multi-spectral imaging system.

Owing to the characteristics of combining spectral and imaging information of HSI, it was possible to conduct pixel-wise analysis and object-wise analysis [24,25]. In this study, the widely-used pixel-wise analysis was presented as PCA score images in Figure 2. The object-wise analysis was performed by using the average spectra of each grape seed to build the discriminant model, through which the classification maps were formed to visualize the sample profiles. For the purpose of assessing the whole quality of grape seeds, a comprehensive study should be conducted in the future. Total flavanol content in 18 samples of one red grape variety (cv. Tempranillo) and 15 samples of one white variety (cv. Zalema) of three replicates was determined by HSI in a previous study [26]. Other chemical constituents measured by reference chemical tests should also be taken into account in future studies. With the aid of programming algorithm and automatic control techniques, an on-line assessment system of whole quality of grape seeds could be developed by taking advantage of HSI.

## 4. Materials and Methods

### 4.1. Sample Preparation

Three grape seed varieties (Hongtizi, Meirenzhi, Jufengxiahei) harvested in 2017 were bought from a commercial seed company (Shuyang Pengyuan horticulture farm, Suqian, Jiangsu, China), and they were recorded as Variety I, Variety II and Variety III, respectively. About 500 g dry seeds of each variety were collected. The seeds were cleaned first, and the damaged seeds were removed. The number of grape seeds for image acquisition was 14,015, 14,300 and 15,042 of each variety, respectively.

### 4.2. Hyperspectral Image Acquisition

#### 4.2.1. Hyperspectral Imaging System

A line-scanning near-infrared HSI system was set up to acquire hyperspectral images of grape seeds. This system consists of an imaging spectrograph, an InGaAs camera, an illumination unit and a conveyer belt. The imaging spectrograph (ImSpector N17E; Spectral Imaging Ltd., Oulu, Finland) covers the spectral range of 874–1734 nm. The high performance camera (Xeva 992; Xenics Infrared Solutions, Leuven, Belgium) is coupled with a camera lens (OLES22; Specim, Spectral Imaging Ltd., Oulu, Finland). The resolution of the camera is 320 × 256 (spatial × spectral) pixels. Two 150-W tungsten halogen lamps (3900e Lightsource; Illumination Technologies Inc.; West Elbridge, NY, USA) were used as the illumination unit to provide a sufficient light source. A conveyer belt driven by a stepper motor (Isuzu Optics Corp., Zhubei, Taiwan) was applied to move the samples. The parameters for image acquisition (i.e., the exposure time of the camera and the moving speed of the conveyer belt) were controlled by a computer equipped with a data acquisition and preprocessing software (Xenics N17E; Isuzu Optics Corp., Zhubei, Taiwan).

#### 4.2.2. Hyperspectral Imaging Acquisition and Correction 

Grape seeds were placed on a black plate without overlapping or connecting with each other. The plate was then put on the conveyer belt for image acquisition. The distance between the camera lens and the plate was set to 11.0 cm, and the exposure time of the camera was set to 2 ms. A total of 256 spectral bands was generated by the spectrograph. The images were acquired by uniformly moving the plate along the X-axis with a speed of 11.5 mm/s. These images were recorded as the raw images (*I*_raw_). In order to reduce the influence of light and noise, the white reference image and the dark reference image needed to be obtained under the same condition as the sample image acquisition. The corrected images could be obtained using the following equation:

(1) $$I_{c}=\frac{I_{\mathrm{raw}}-I_{\mathrm{dark}}}{I_{\mathrm{white}}-I_{\mathrm{dark}}}$$

where *I*_c_ is the corrected hyperspectral image, *I*_raw_ is the raw hyperspectral image and *I*_dark_ and *I*_white_ are the dark and white reference images, respectively. The standard dark reference image was obtained by turning off the light source and completely covering the camera lens with an opaque cap. The standard white reference image was obtained by recording the spectral image of a white Teflon bar of high-reflectance (approximately 100%).

### 4.3. Data Analysis

#### 4.3.1. Data Extraction and Preprocessing

**Figure 5:**
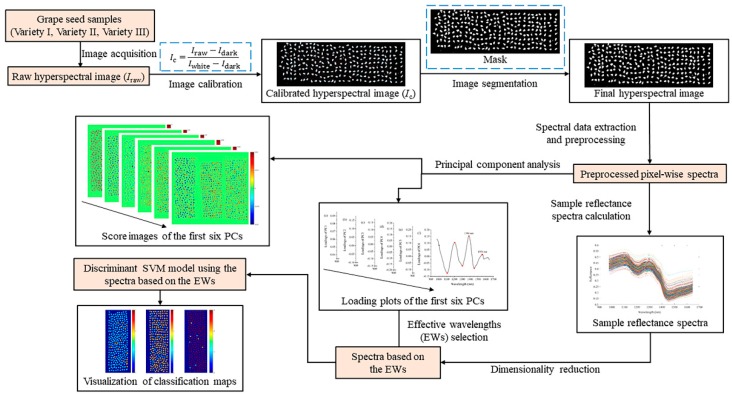
Main steps for analyzing hyperspectral images and establishing the discriminant model for grape seed varieties. EW, effective wavelength.

The procedure of analyzing hyperspectral images and establishing the discriminant model for grape seed varieties is presented in Figure 5. Image segmentation was carried out on the corrected hyperspectral images. This procedure began with creating a mask to segment the grape seed samples from the background. The gray-scale image at 1119 nm where the reflectance difference between the background and the sample region reached the maximum value was used to form a mask. The mask was formed by setting the numerical value of all background pixels to 0 and all pixels within the samples to 1. By multiplying the binary value of pixels in the mask and the corresponding reflectance value in grayscale images, the pixel-wise spectra within the samples were kept, while the spectra belonging to the background were removed. The region of interest (ROI) was defined as each individual sample region segmented from the background.

Appropriate data preprocessing is beneficial for multivariate classification. It aims at reducing variation sources that carry irrelevant information. The beginning and the end of the spectral data contained considerable random noise, so the middle 200 bands from 975 nm–1646 nm were used for further analysis. For the purpose of reducing the random noise in the pixel-wise spectra of the ROIs, a typical pre-processing method, wavelet transform (WT), was used for smoothing. The wavelet function of Daubechies 7 with a decomposition level of 3 was used. Sample reflectance spectra were obtained by calculating the average value of the pixel-wise spectra of all pixels within the ROI.

#### 4.3.2. Sample Set Split

In this study, for each grape seed variety, fifty-five hyperspectral images were acquired. Before building discriminant models based on recognition techniques, classification rules should be settled first. A calibration set with known classes served to create the classifier, while a prediction set was used to validate the performance of the established model. In this study, grape seed samples in fifty-four images were divided into the calibration and prediction sets, as a division ratio of 2:1. As a result, grape seed samples in thirty-six images were chosen as the calibration set, containing in total 28,290 samples, including 9120, 9309 and 9861 samples of Variety I, Variety II and Variety III, respectively. The prediction set contained in total 4640, 4724 and 4903 samples of Variety I, Variety II and Variety III, respectively. In addition, for each variety, samples in another image were used for external validation and visualization. The number of sample was 255, 267 and 278 of Variety I, Variety II and Variety III, respectively.

#### 4.3.3. Principal Component Analysis

PCA is a commonly-adopted method in multivariate statistical analysis that reduces the dimension and extracts the useful information of the data matrix [27]. It replaces the original variables with a group of new variables called the PCs, which are a linear transformation of the original variables. The first PC extracts the maximum variance from the original spectral data. Then, it looks for the second PC that explains the maximum variance of the remaining variables, and so on. The transferred PCs are orthogonal to each other and are arranged in descending order of explained variance. The first few PCs often cover the most useful information of the original data matrix. Scores of the PCs help to identify the presence of groups or patterns in the data. Loadings of the PCs are the transform matrix between the original variables and the independent PCs, which reflect the contribution of the original variables [28]. In this study, the first few PCs with the greatest contribution of total variance were used to draw the score images of the pixels within the ROIs to identify the pixels with similar spectral features. PCA loadings were used to select the EWs.

#### 4.3.4. Discriminant Method

SVM is a commonly-used supervised pattern recognition method based on structural risk minimization [29]. SVM intends to maximize the margin among classes and minimize the classification error in the meantime [30]. It works by mapping the origin data of a low dimension space into a higher dimension space and constructs a separating hyperplane to realize linear classification. The computational complexity will be effectively reduced by introducing a kernel function [29]. A popularly-used kernel function, the radial basis function (RBF), was applied in this study. For SVM models using RBF as the kernel function, parameters, such as the penalty coefficient (c) and kernel width (g), should be determined, and these parameters were set by a simple grid-search procedure in this study.

#### 4.3.5. Software

ENVI 5.1 (ITT Visual Information Solutions, Boulder, CO, USA) was used to create the mask for segmenting grape seed samples in hyperspectral images without irrelevant background. MATLAB R2017b (The MathWorks, Natick, MA, USA) was used to extract and preprocess the pixel-wise spectral data from hyperspectral images. Multivariate analysis methods, including transformation of PCA for pattern recognition and wavelength selection, establishment of SVM models for variety discrimination and visualization of the predicted results of the single grape seed in classification maps were also implemented with MATLAB R2017b.

## 5. Conclusions

This study was carried out to evaluate the feasibility of using an HSI technique in the NIR spectral region (874–1734 nm) combined with multivariate analysis methods for non-destructive, rapid and accurate variety discrimination of grape seeds. To promote the robustness and reliability of the model, a large number of grape seed samples of three varieties was collected to form the final dataset. Grape seed samples in fifty-four hyperspectral images of each variety were split into the calibration and prediction sets at a ratio of 2:1. In addition, one image of each variety was chosen as the external validation image to assess the prediction ability of the established discriminant model and for visualization. According to the score images from PC1–PC6, grape seed samples of different varieties could be roughly identified, but establishing qualitative discriminant models for accurate classification was necessary. Since hyperspectral images contained a large amount of data with redundancy and collinearity which were difficult to deal with, loadings of the first six PCs were used to recognize the EWs. The spectra based on the EWs were extracted to serve as the independent variables to build the SVM model. The discriminant results were satisfactory, with calibration accuracy of 94.3% and prediction accuracy of 88.7%. Further validation was carried out by using the external validation images. The prediction results of the variety of each single grape seed were visualized in the classification maps, which showed great potential for large-scale detection in the modern seed industry. Furthermore, the implementation of wavelength selection reduced the detecting bands to 10, which saved the computational cost of the discriminant model and sped up the prediction process. The overall results indicated that it was convenient to utilize this non-destructive technique to predict the variety of each single grape seed and visualize the corresponding variety in the classification maps. The factors influencing the detection of seeds’ quality can be explored by the HSI technique in the future. This research provides guidance for developing on-line detection systems of grape seed quality.

## Figures and Tables

**Table 1:** Discriminant results of grape seed samples of three varieties by the support vector machine (SVM) model.

	Calibration Set (28,290 Samples)				Prediction Set (14,267 Samples)			
	1	2	3	Accuracy	1	2		Accuracy
1	8355	742	23	91.6%	4020	592	28	86.6%
2	760	8495	54	91.3%	947	3767	10	79.7%
3	11	23	9827	99.7%	27	13	4863	99.2%
Total				94.3%				88.7%
